# The National Composite Index for Family Planning (NCIFP): Results and Methodological Issues

**DOI:** 10.12688/gatesopenres.13464.1

**Published:** 2022-01-07

**Authors:** Rebecca Rosenberg, John Ross, Karen Hardee, Imelda Zosa-Feranil

**Affiliations:** 1 M&E and Advocacy, Avenir Health, Glastonbury, CT, 06033, USA; 2 Independent Demographic Consultant, New Paltz, NY, 12561, USA; 3 What Works Association, Arlington, VA, 22207, USA

**Keywords:** Family planning, Global, Rights-based programming, Program effort, Measurement, Monitoring and Evaluation

## Abstract

**Background: ** The “ FP2020 Global Partnership” signaled a shift to broader, rights-based approaches to family planning programs, and the National Composite Index for Family Planning was developed as part of related measurement efforts.

**Methods:** In each country 10-15 experts on the family planning program completed a 35-item questionnaire, first in 2014 in 89 countries, and in 2017 in 84 countries. Data were entered in Excel, with checks for consistency and data quality. The total score, and scores for each of 5 dimensions of effort are averages across the 35 indicators. Analytic techniques included cross-tabulations, graphical and correlation approaches.

**Results: **The average total score for all countries in 2017 was 64 of the maximum of 100 of effort. Sub-regions differed: Anglophone and Francophone sub-Saharan Africa (SSA) scored highest in the total score and across all 5 dimensions. Next in order came Latin America and the Caribbean, Asia, the Middle East and North Africa, and Eastern Europe and Central Asia. Despite large differences in scores, the sub-regions followed similar profiles across the 35  indicators.

The long term rise in the basic family planning effort scores continued, extending the series from surveys approximately every five years beginning in the 1980s. The highest score reached was for the strategy dimension, but the others were close. Their relative levels remained essentially the same as in the 2014 survey.

NCIFP scores correlated positively with modern contraceptive use in both the sub-Saharan Africa (SSA) and non-sub-Saharan Africa (non-SSA) countries, but the relationships were stronger for SSA. Access to long-acting and permanent methods (LAPMs) was accompanied by greater LAPM use and modern method use.

**Conclusion: **Repeated surveys in most developing countries show improvements in family planning effort, though unevenly, by 35 indicators and across regions.

## Introduction

For many decades, there has been a sustained interest in measuring the nature and strength of family planning (FP) programs, to understand how efforts change over time and how they relate to key FP outcomes. The Family Planning Effort Index (FPE) has been used to measure the effort of large-scale FP programs periodically since 1972, providing cross-national comparisons and tracking progress over time ^1– 9^. Four key components of FP program input are covered: policies, services, evaluation and access. The policy component pertains to the enabling environment, including such items as support from in-country leaders, with defined objectives and explicit budget allocation for FP. The services component concerns items such as the use of community-based distribution, whether facilities are adequately staffed, and the degree of training and supervision. The evaluation component assesses not only the extent of data collection but also the use of data to inform decisions. Finally, the access component captures the extent to which various contraceptive methods and method-specific services are available to the entire population ^8^. Existing studies have used FPE scores to monitor trends in the effort of FP programs over time, compare program strength at the sub-national level, and measure the association between FP program effort and FP indicators, including contraceptive use, contraceptive method mix and fertility. Most of these studies have found a positive relationship between FPE scores and FP indicators: as FP program effort improves, fertility levels tend to be lower ^1, 6^, and contraceptive use tends to increase ^9^.

In July 2012, government leaders, donors, researchers, and civil society groups met at the Family Planning Summit in London to launch the Global FP2020 Partnership, commonly known as FP2020. It emphasized an enabling environment for FP through program development and implementation grounded in rights-based programming ^10^. It aimed to empower all individuals to act on their fertility choices through improved access to high-quality reproductive health information, education, and services, free from discrimination, coercion and violence. Building on the FPE and FP2020’s Rights and Empowerment Principles, ^a^ the National Composite Index for Family Planning (NCIFP) was developed to support FP2020 measurement efforts, focusing on rights and equity: both fundamental pillars of FP2020. In 2014, the NCIFP questionnaire was added at the end of the FPE questionnaire, so data were gathered on both instruments at the same time in all countries. The intention was not to replace the FPE but to build on the standard FPE questions, adding items to capture areas not fully covered by it, particularly issues related to rights (quality and accountability) and equity.

The NCIFP is comprised of five dimensions: Strategy, Data, Quality, Equity and Accountability. ^b^ The Strategy and Data dimensions capture many of the same items as the FPE. The quality dimension measures whether quality of care indicators are monitored, and whether there are structures in place to support quality services. The equity dimension focuses on issues in both policies and programs related to discrimination, efforts to reach under-served groups and wide-spread access to methods. Lastly, accountability focuses on monitoring and ensuring informed choice and voluntariness, while avoiding coercion and denial of services based on non-medical grounds.

This paper presents the main findings of the 2017 NCIFP along with comparisons to the 2014 findings to explore changes over time. The results show improvements in the policies and program implementations across all five dimensions of effort: Strategy, Data, Quality, Equity, and Accountability. NCIFP scores offer guidance to inform future policy judgements and resource allocations. At the country level, decision-makers can review the scores for specific items to identify areas for potential improvements.

The participating countries in the two rounds are listed in Table 1. 

**Table 1.** Countries by Regional Grouping (countries with data for 2014 are in bold font).

2017 Countries by Regional Grouping (countries with data from 2014 are in bold)					
Asia (ASIA)	Latin America and the Caribbean (LAC)	Middle East/ North Africa/ (MENA)	Anglophone Sub-Saharan Africa (SSAF-A)	Francophone Sub- Saharan Africa (SSAF-F)	Eastern Europe and Central Asia (EECA)
**Afghanistan** **Bangladesh** Bhutan **Cambodia** I **ndia** Lao PDR **Malaysia** Mongolia Myanmar Nepal Pakistan Papua New Guinea Philippines **Sri Lanka** Solomon Islands **Timor-Leste** **Viet Nam**	**Bolivia** Colombia **Dominican Rep.** **El Salvador** **Guatemala** **Haiti** **Honduras** **Jamaica** **Mexico** Nicaragua **Panama** **Peru**	**Egypt** **Iraq** **Jordan** **Morocco** State of Palestine	**Cameroon** **Eritrea** **Eswatini** **Ethiopia** **Gambia** **Ghana** **Kenya** **Lesotho** **Liberia** **Malawi** **Namibia** **Nigeria** **Rwanda** Sierra Leone Somalia **South Sudan** **Tanzania** **Uganda** **Zambia** **Zimbabwe**	Burkina Faso **Burundi** Central African Rep. (CAR) **Chad** **Congo** **Cote d’Ivoire** **DR Congo** Guinea **Guinea-Bissau** **Madagascar** **Mali** **Mauritania** **Mozambique** **Niger** Sao Tome & Principe **Senegal** **Togo**	**Armenia** Azerbaijan * **Georgia** **Kazakhstan** **Kyrgyz Republic** **Moldova** **Romania** **Russia** **Tajikistan** **Turkmenistan** **Ukraine** **Uzbekistan**

*removed from analysis due to data quality concerns.

## Methods

The NCIFP uses a key informant approach, identifying experts in each country who have a close acquaintance with the family planning program. Data collection in each country was managed by a local consultant who was familiar with the national program and could identify people who could gauge the effort levels in its various features. In each country 10–15 local respondents completed the questionnaire; respondents were working in four different capacities: inside the FP program, in local NGO organizations, in local academic or research organizations, and resident staff of international agencies. The objective of the surveys was to represent the 69 FP2020 priority countries (the world’s poorest in 2012) and around 30 other countries in the low-and-middle-income group.

The first round of the NCIFP was conducted in 2014 in 89 countries. In 2017, a second round of data collection took place in 84 countries to enable monitoring of efforts over time, and to improve certain features of the methodology. ^c^ A total of 71 countries participated in both the 2014 and 2017 rounds. Table 1 shows a list of participating countries, by region.

The 2014 round of the NCIFP was comprised mostly of yes/no questions along with some 1–10 scale questions. However, data collection revealed several difficulties related to asking yes/no questions. First, the score for most questions ended up simply representing the percent of respondents who said yes. Additionally, for some questions, a clear cut yes or no answer was not feasible because the question asked about multiple issues, or the answer fell into an intermediate place between a simple “yes” or “no” response. To address these issues, 1–10 scale responses were added after every yes/no question in the 2017 round to allow for finer gradations.

The NCIFP provides 35 scores to measure national efforts for family planning that fall into the five dimensions of Strategy, Data, Quality, Equity, and Accountability. To allow for trend analysis between 2014 and 2017, the scores in this paper are based on the 2014 approach of yes/no items but with some 1–10 scaled items. The full list of scores with information on modifications between the two rounds is available in Table 1 of the *Extended data* for this paper ^11^. Where the 2014 and 2017 results are compared directly in the report they pertain only to the 71 countries participating in both rounds.

Data were entered in Microsoft Excel (RRID:SCR_016137), with checks for consistency and data quality ^12^. Responses were averaged to obtain a country score for each question. The total score, and scores for each dimension were calculated by averaging across the individual questions. Where appropriate, total and regional averages were weighted by the number of women of reproductive age (15–19 years) in each country ^d^ and reflect the diversity of population sizes. For example, the Asia region accounts for 57% of women, and within it, India has about two-thirds of the regional total. The numbers and percentage distributions by region and by country within each region are available in Table 2 of the *Extended data*.

Analytic techniques include cross-tabulations, graphical and correlation approaches. Methodological issues of special interest are discussed in detail in the final section of the article.

## Results

### Global and regional results

**Figure 1.**
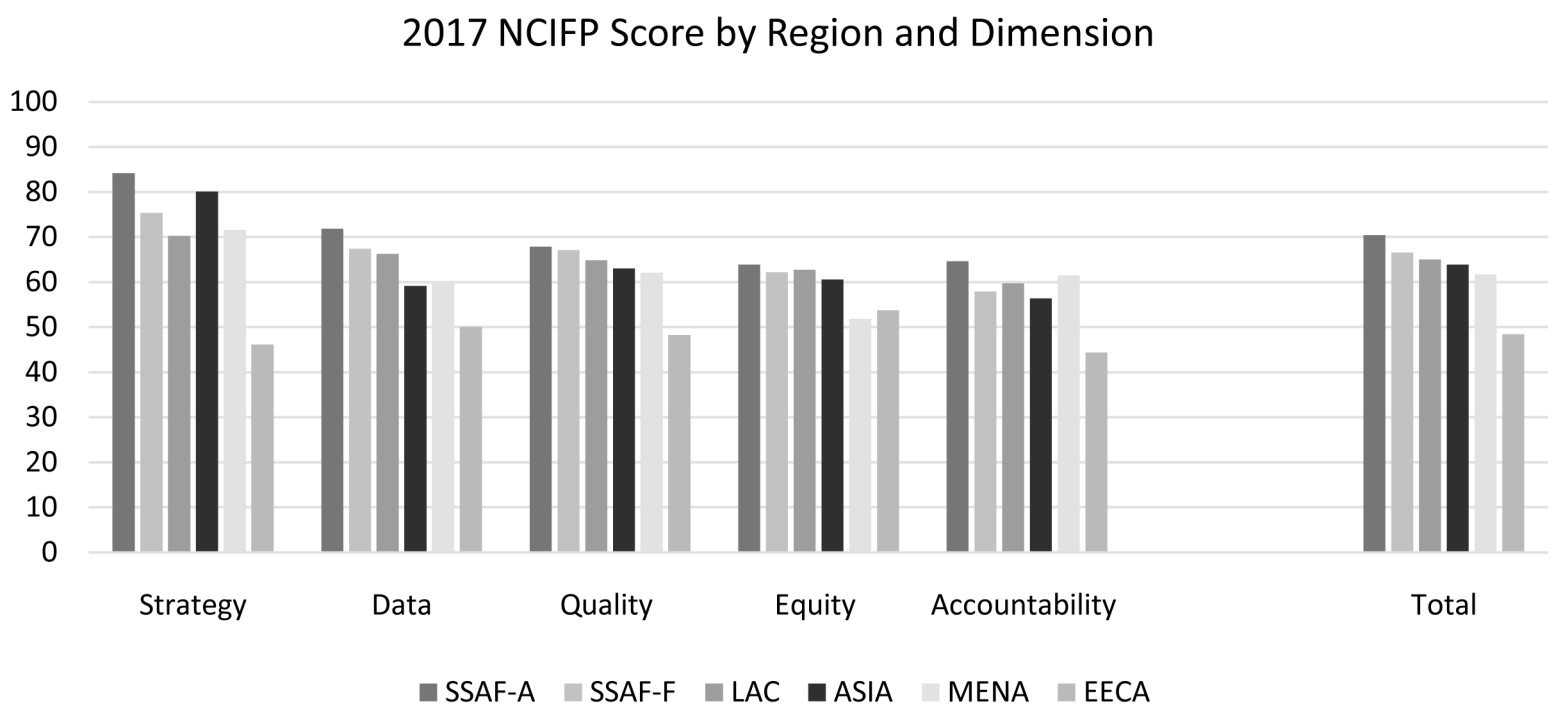
2017 NCIFP by Region and Dimension (weighted).

In 2017, the average total score was 63.9, about two-thirds of the maximum of 100.0. This varied from 57.3 to 76.6 across the five dimensions of effort. The 2017 scores by dimension and region are displayed in Figure 1. Anglophone sub-Saharan Africa (SSAF-A) scored the highest across all dimensions. Rankings for the other regions varied, except that the Eastern Europe and Central Asia (EECA) region fell well below the others on all dimensions except equity. In every region except EECA, Strategy was the highest scoring dimension.

### Trends

How much did the scores improve between 2014 and 2017? Because of methodological changes between the two rounds, the trends can be assessed only for score items that were worded the same in both surveys. These are the “FPE” items that were carried over from the long-term series conducted approximately every five years starting in the 1987s. By dimension these are:

Strategy:

High level support for the family planning program
Laws and regulations favor contraceptive imports or local manufacture

Data:

Extent to which record keeping and feedback of results are adequate
Extent to which program statistics, surveys, and studies are used to measure progress
Extent to which managers use research and evaluation findings to improve program.

Quality:

Extent to which the logistics and transport systems are sufficient for stocks and equipment
Extent to which training programs for staff are adequate for effective job performance
Extent to which the system of supervision is adequate, with corrective or supportive actions
Extent to which clients adopting sterilization are informed that it is permanent
Extent to which the entire population has ready and easy access to IUD removal
Extent to which the entire population has ready and easy access to implant removal.

Equity:

Extent to which underserved areas are covered by CBD programs to distribute contraceptives
Extent to which the entire population has ready and easy access to the following (7 separate questions): female sterilization, female sterilization, IUD, implant, injectable, pill, and condom.

(The fifth dimension, Accountability, was created with entirely new questions.)

**Figure 2.**
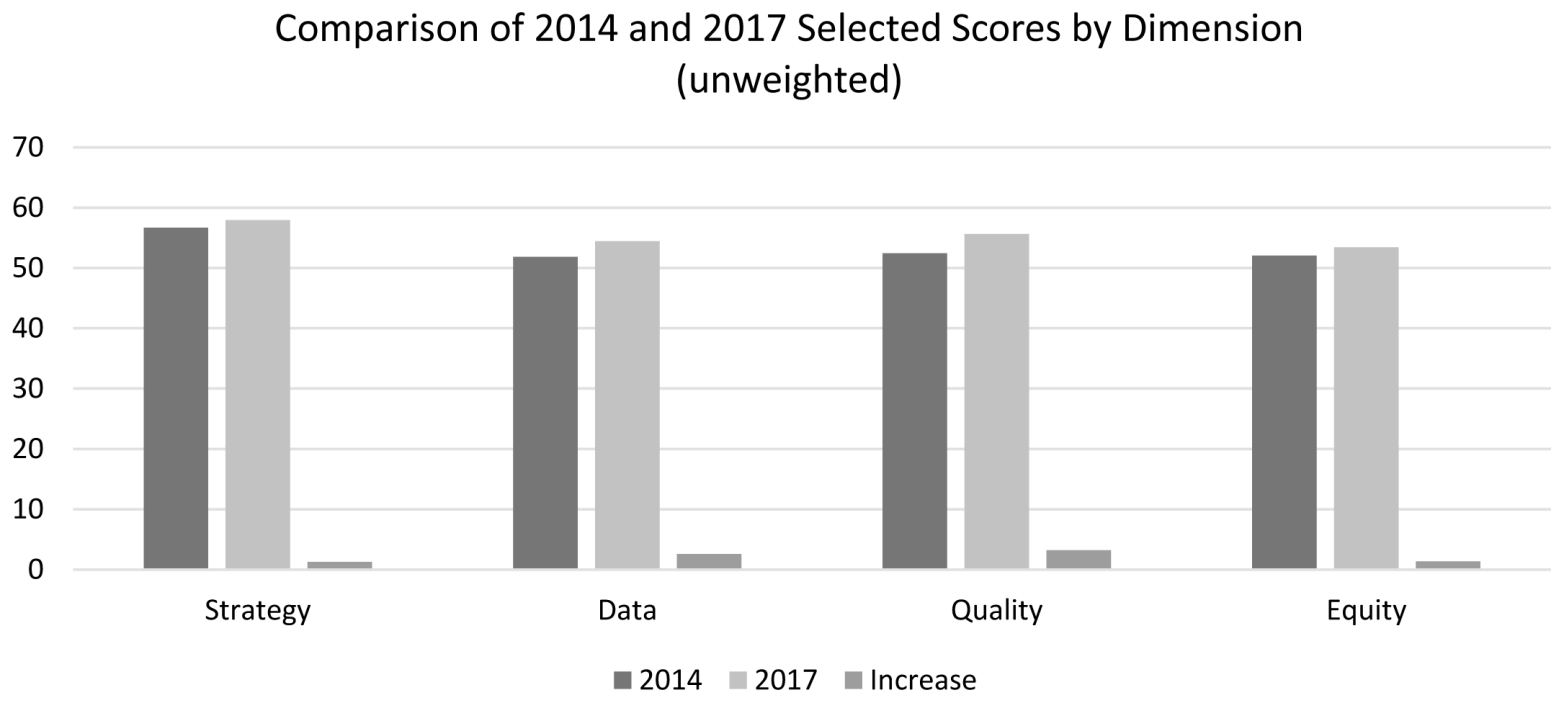
Comparison of 2014 and 2017 Scores for FPE Items, by Dimension.

Overall, these scores improved over time, as they did in the inter-survey intervals in previous FPE surveys. Figure 2 presents the 2014 and 2017 results for the 13 items above. Overall, the scores ranged in the mid-50s, with the highest for Strategy in 2017 at 57.9 and the lowest two for Data and Equity at 51.8 and 52.0 in 2014.

Against those levels, the increases ranged from 1.3 to 3.2 across the four dimensions, or roughly 2.2%, 5.0%, 6.1%, and 2.6% for Strategy, Data, Quality and Equity, respectively. The score change in Data between 2014 and 2017 suggests an increased emphasis on program monitoring through data. Nevertheless, the 2017 level is just above half of programs with adequate record keeping and data use.

Patterns have remained largely the same with Strategy being the highest scoring dimension by a small margin in both years. These results are shown by individual scores in Figure 4 and Figure 5, first for levels and second for changes; note that under Equity access to the seven contraceptive methods is combined for long term *versus* short-term methods.

### Country variations

**Figure 3.**
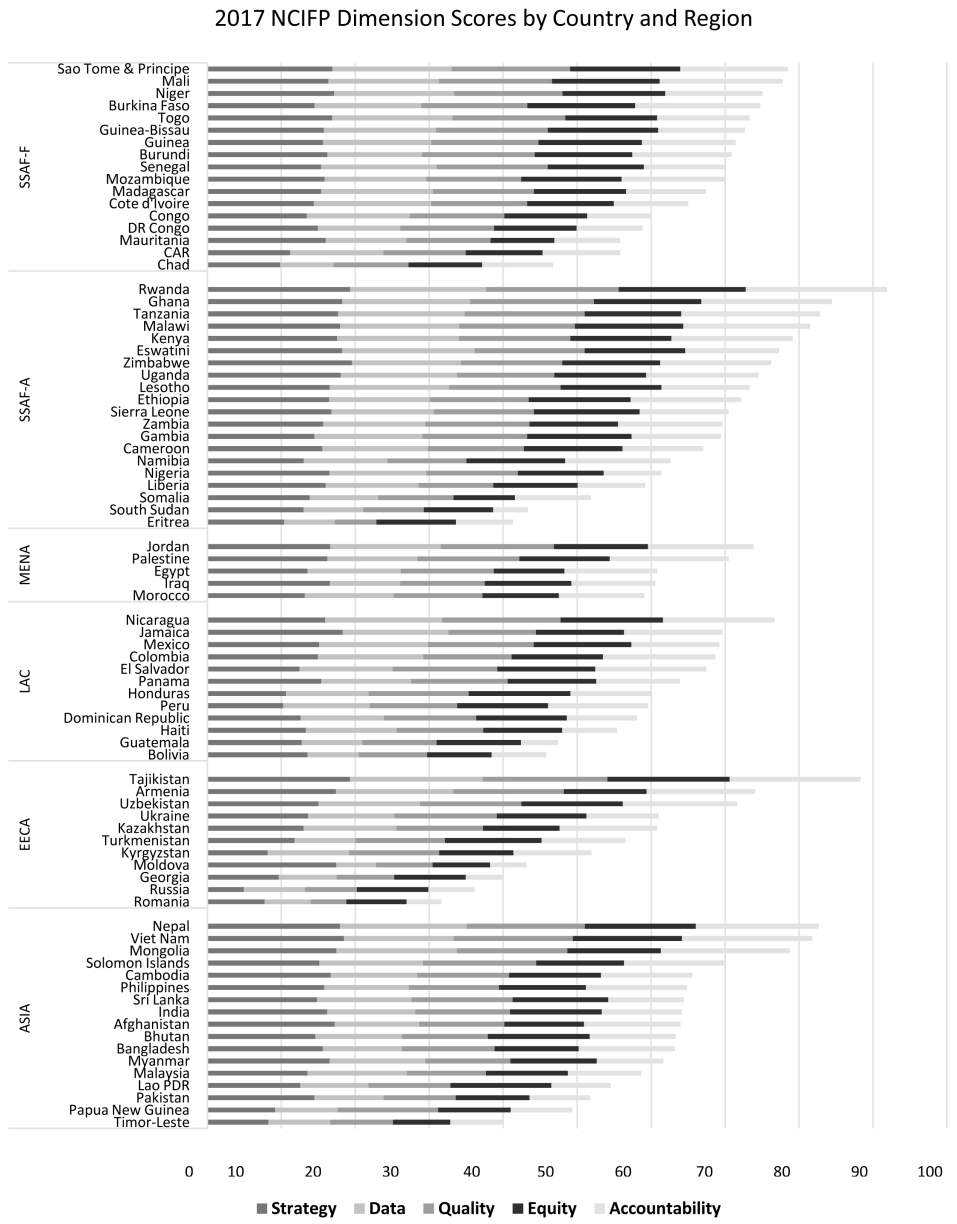
2017 NCIFP Dimension Scores by Country and Region.

Score variations by country and dimension are shown in Figure 3, arranged by the total score within each region in 2017. There is a substantial country variation in scores within each region ^e^. EECA has the widest range of total scores, with Tajikistan as the highest score (87.9) and Romania as the lowest (30.0). Countries in the Middle East and North Africa (MENA) have the smallest range of total scores, from 59.5 for Morocco to 74.7 for Jordan. Among all regions, Rwanda has the highest score (91.6) and Romania the lowest.

The relative bar lengths across the five dimensions within the shrinking total lengths are fairly consistent in the two African regions. That is, the relative space given to each color does not change much as the total bar shortens. That suggests that where total effort is less the relative stress allocated to each dimension remains about even.

The pattern of fairly even shares of attention appears also in MENA and Asia, but less so in Latin America and the Caribbean (LAC) and EECA.

Consistent with the previous figures, this again shows that for most countries, the highest dimension score is for Strategy, and the lowest is for Accountability.

### Patterns for the 35 scores by region

**Figure 4.**
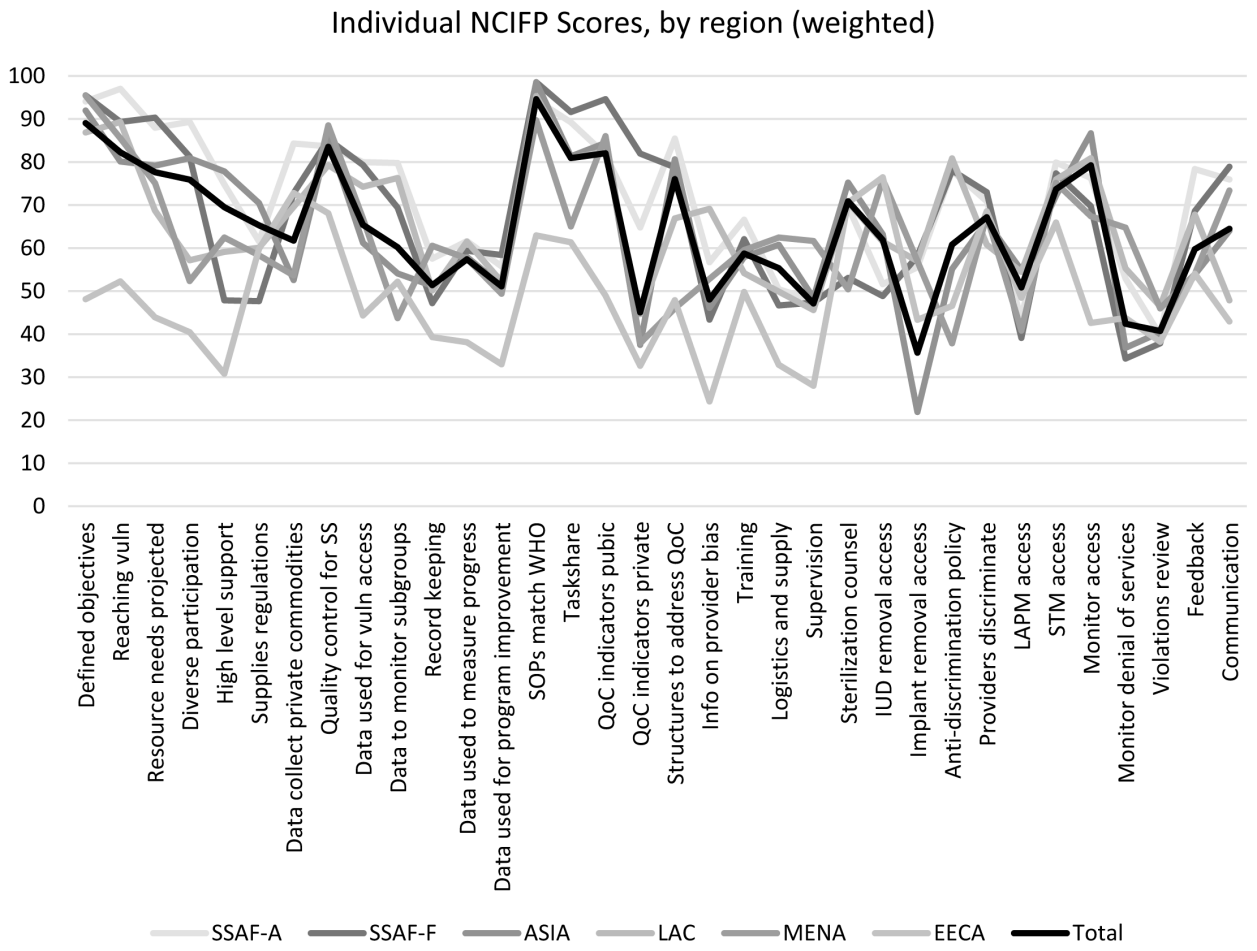
Individual NCIFP Scores According to Region (weighted), 2017.

A decided commonality exists across regions in the uneven profile of effort across the 35 scores. The regional lines, to a remarkable degree, move up and down together. The agreements emerge despite the large disparities in regional characteristics and programs. Each regional line comes from the average across its member countries, so the disparities in both country and regional patterns are over-ridden by the nature of each score. For example, it is much easier in most countries, under Strategy, to create a set of objectives (at the left) than to obtain high level support (at the right). Under Equity, it is simpler to declare an anti-discrimination policy than to deploy a CBD program. Therefore, the highs and lows in Figure 4 (total line) appear to reflect the essential “ease” by which countries can score on each kind of effort. The countries clearly exert stronger efforts on some of the 35 items than others, but they face dissimilar barriers across the items. 

For four of the regions (SSAF-F, Asia, LAC and EECA), the highest scoring item was a measure of Quality: “Are FP Standard Operating Procedures in line with WHO and used for determining areas of need for quality FP improvement?”. In SSAF-A, the highest scoring item was a measure of Strategy: “Does the national family planning action plan include objectives to reach the poorest and most vulnerable groups with quality FP information and services?”. MENA’s highest scoring item was also related to Strategy: “Does the national FP action plan include defined objectives over a 5 to 10-year period, including quantitative targets?”. There was less consistency in terms of the lowest scores by region. Lowest scores per region were as follows:

- SSAF-A: Are violations reviewed on a regular basis? (Accountability)
- SSAF-F: Does the government have mechanisms in place for reporting instances of denial of services on non-medical grounds or coercion? (Accountability)
- Asia: Extent to which entire population has ready access to implant removal (Quality)
- LAC & MENA: Extent to which areas of the country not easily serviced by clinics or other service points are covered by CBD programs for distribution of contraceptives. (Equity)
- EECA: Does government collect information related to informed choice and provider bias? (Quality)

The lowest scoring item for SSAF-A and SSAF-F fell within the Accountability dimension. For Asia and EECA, the lowest scoring item was in the Quality dimension, and for LAC and MENA, the lowest scoring item fell within the Equity dimension. None of the lowest scoring items fell within the Strategy dimension. 

**Figure 5.**
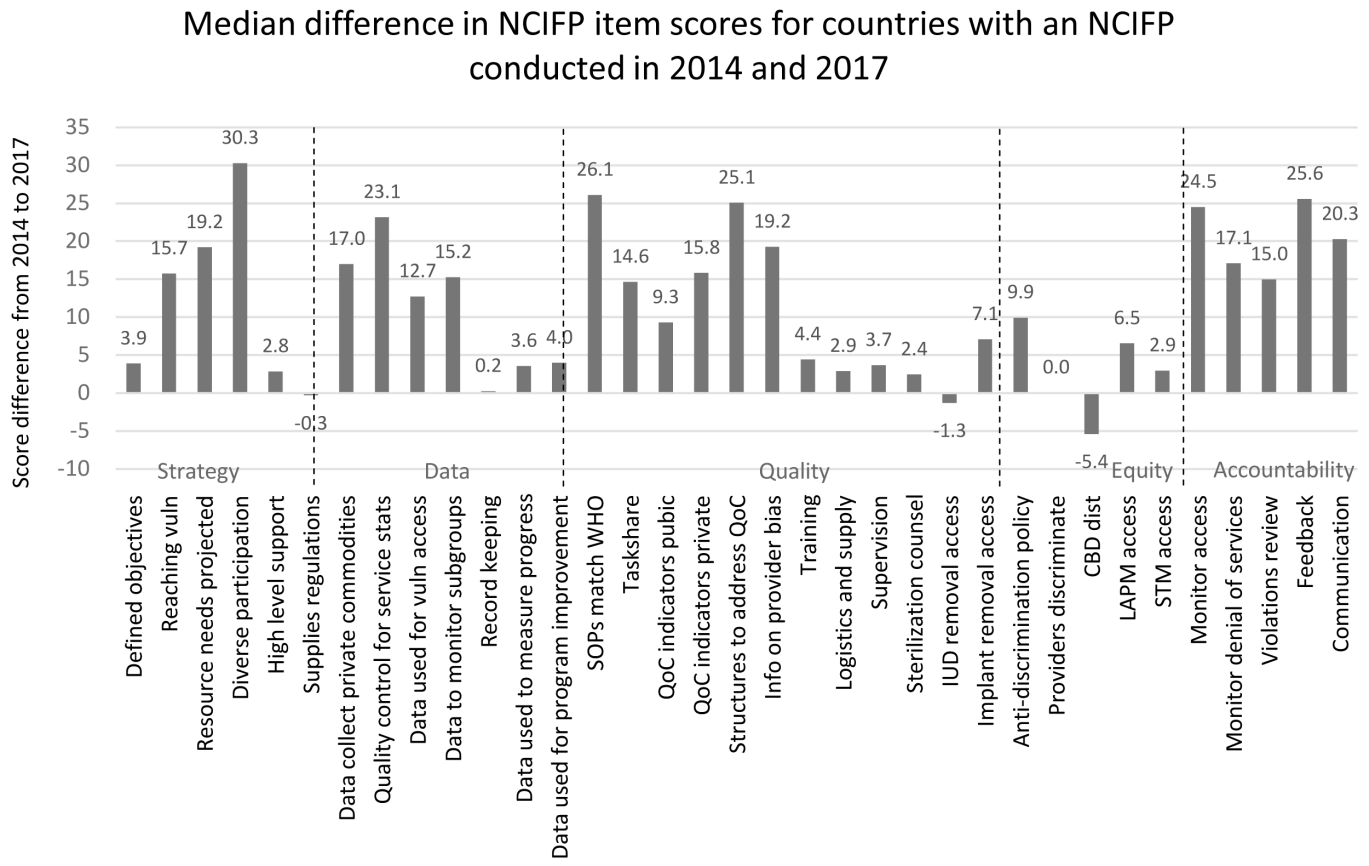
Gains in the Scores between 2014 and 2017.

Gains over time occurred in nearly all of the 35 items, as shown in Figure 5, based on the median scores across countries (those with data in both years). Among these countries, the largest gain was for the item “Does the National Family Planning Action Plan include a mechanism and funding to support meaningful participation of diverse stakeholders?” (30 points). For three items, the difference between 2014 and 2017 was negative: “Extent to which import laws and regulations facilitate the importation of contraceptive supplies or extent to which contraceptives are manufactured locally” (−0.3 points), “Extent to which the entire population has ready and easy access to IUD removal” (−1.3 points) and “Extent to which areas of the country not easily serviced by clinics or other service points are covered by CBD programs for distribution of contraceptives (especially rural areas)” (−5.4 points). Half of the 35 items had an unweighted median point increase of 10 or more points. Every item in the Accountability dimension had a median point increase of at least 15 points. 

### Relationship of contraceptive use to the NCIFP scores

**Figure 6.**
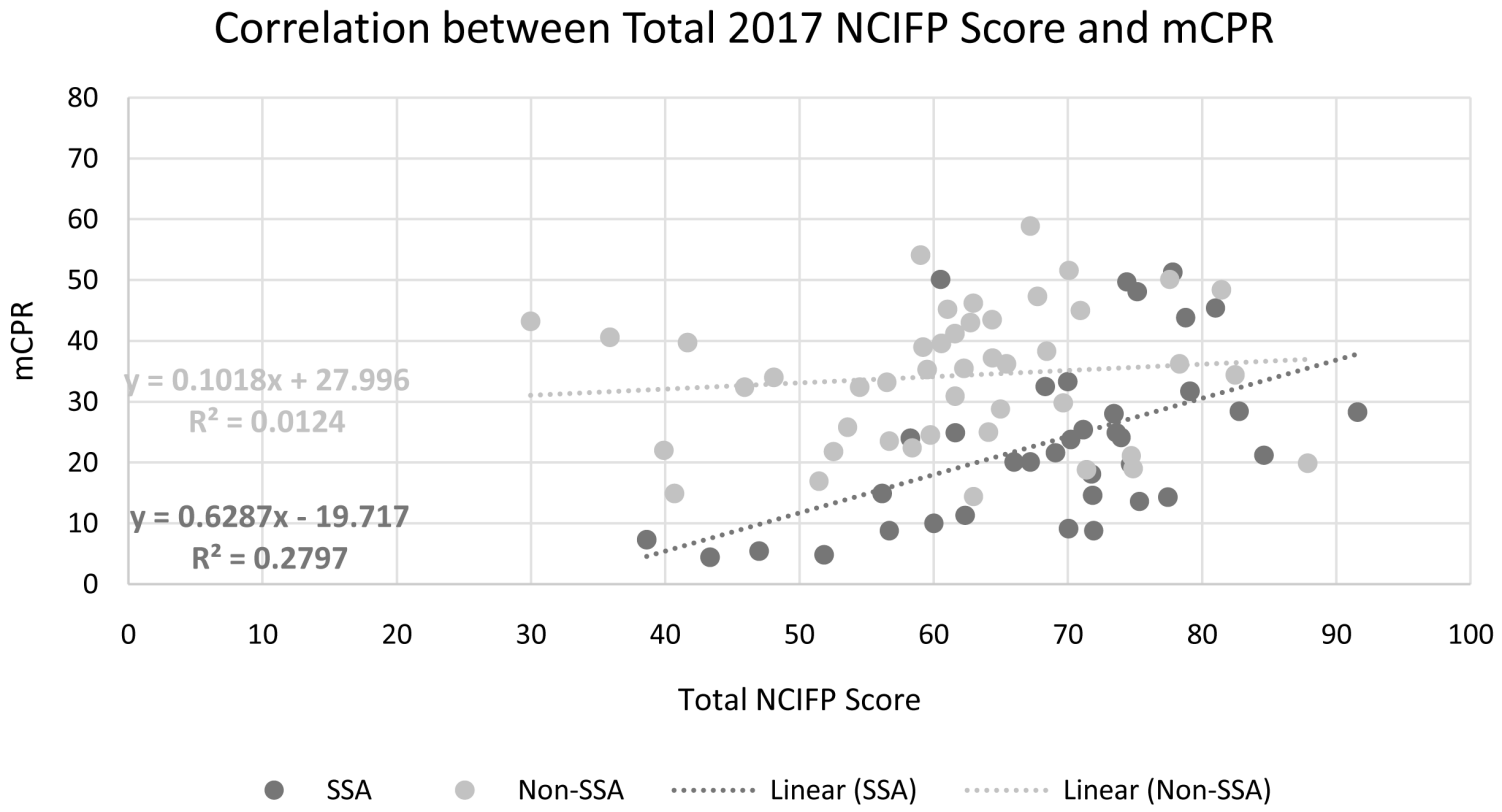
Total 2017 NCIFP Score and mCPR, by regional groupings.

To examine the relationship between contraceptive use and NCIFP, we had to combine the smaller regional groupings ( *i.e*., SSAF-A, SSAF-A, Asia, LAC, MENA, EECA) into larger regional groupings (sub-Saharan Africa [SSA] and non-sub-Saharan Africa [non-SSA]) to allow for more data points per regional grouping. Higher modern contraceptive use was correlated with the NCIPF scores in both the SSA and non-SSA regional groupings ( Figure 6), but the relationship was much stronger for SSA, which is of special interest as the region with the lowest contraceptive use, the highest fertility rates, and the highest levels of infant and child mortality. In SSA, a ten-point increase in the total score is accompanied by a 6-point increase in mCPR, but by a mere 1-point increase in Non-SSA. The average mCPR ^f^ across SSA countries is 23.4%, with a minimum of 4.4% (South Sudan) and a maximum of 51.3% (Eswatini). Among Non-SSA countries the average mCPR is higher by half, at 34.2%, with a minimum of 14.4% (Afghanistan) and a maximum of 58.9% (Colombia).

A similar analysis was done to explore the relationship of the total fertility rate (TFR) to the NCIFP, which found a much weaker correlation than for the mCPR. That is not entirely surprising as the TFR responds to several determinants, including the use of traditional methods, and is not as directly impacted by programmatic efforts as mCPR.

Correlations by the five NCIPF dimensions give further evidence of closer relationships to contraceptive use in the SSA region ( Table 2). By the total score, the correlation is r=0.53 in SSA and only r=0.11 in Non-SSA. There is little variation across dimensions, with SSA Data dimension having the highest coefficient (0.55) and Quality the lowest (0.41).

**Table 2.** Correlation between 2017 Dimension scores and mCPR by regional grouping.

	Correlation between Dimension Scores and mCPR (all women)
	**mCPR: SSA Countries**
***Total Score***	r=0.53
***Strategy***	r =0.50
***Data***	r =0.55
***Quality***	r =0.41
***Equity***	r =0.47
***Accountability***	r =0.50
	**mCPR: Non-SSA Countries**
***Total Score***	r =0.11
***Strategy***	r =0.02
***Data***	r =0.10
***Quality***	r =0.09
***Equity***	r =0.26
***Accountability***	r =0.15

In Non-SSA countries, the mCPR is very weakly correlated with the dimension scores. The Equity dimension had the highest correlation coefficient (0.26) and Strategy the lowest (0.02).

### Relation of contraceptive use to access

Among the 35 scores, two are related to access, one for long-term and one for short-term methods. The items were scored from 1 to 10, where 10 meant full access. They read as “Extent to which the entire population has ready access to long-acting and permanent methods (LAPMs)” and “Extent to which the entire population has ready access to short-term methods (STMs).” Scores for access to STMs averaged 74.4 across all countries, reflecting easy access to some of these methods, particularly condoms. Scores for access to LAPMs, however, averaged only 44.2 across all countries. These methods, including sterilization, IUDs and implants are entirely clinical, which tends to constrain use in many countries in SSA and elsewhere. The seven regions differed little around the averages.

Interestingly, the correlation between access and use for LAPMs was a high (r=0.58) but that for STMs was a low (r=0.15). ^g^ Perhaps one reason for the disparity is that use of the long-term methods, all requiring clinical visits and procedures, requires more effort by the family planning programs and makes for a closer connection, though other dynamics are likely involved.

While it is encouraging that access to LAPMs is improving, and that access as measured by the NCIFP appears to be correlated with use, it is also important to note access to implant *removal* was one of the lowest-scoring items in the quality dimension (50.5 in 2017) and the item on access to IUD *removal* declined 1.3 percentage points from 2014 to 2017. Access to removal is a critical component in the right to choose when to have a child, so it is imperative that programs ensure that access to removals keeps pace with access to long-acting methods.

### Relation of use by youth to selected NCIFP indicators

The use of modern methods by youth can be related to two items in the Equity dimension that concern policies and programs affecting young people. These pertain to the extent to which service providers do not discriminate against youth, and the extent to which policies are in place to prevent discrimination towards youth. These scores were paired to DHS data on the percent of sexually active youth (aged 15–24 years) currently using a modern method ^h^ for the 17 countries with data from both sources. No association between the youth equity score and mCPR among sexually active youth (ages 15–24 years) was found. These results reflect the presence of other determinants and the disparate set of countries for which data were available. Freedom from prejudices eases one barrier to contraceptive use for youth, but other constraints are also important.

## Further methodological issues

Going beyond the basic features presented in the initial Methodology section, issues of special interest are considered below.

### Yes/no responses versus the 1 to 10 scale

In 2014, the score for most questions was simply the percent of respondents who said yes. The yes/no questions did not allow for gradations in the response; additionally, for some questions a clear cut ‘yes’ or ‘no’ answer was problematic because the question asked about multiple issues. 

In 2017, to allow for nuances in the rating, respondents choosing "yes" for an item were asked to rate it on a scale from 1 to 10, where 1 indicated the effort was non-existent, equivalent to having responded “no”. Then 2 represented very weak effort and 10 an extremely strong effort.

**Figure 7.**
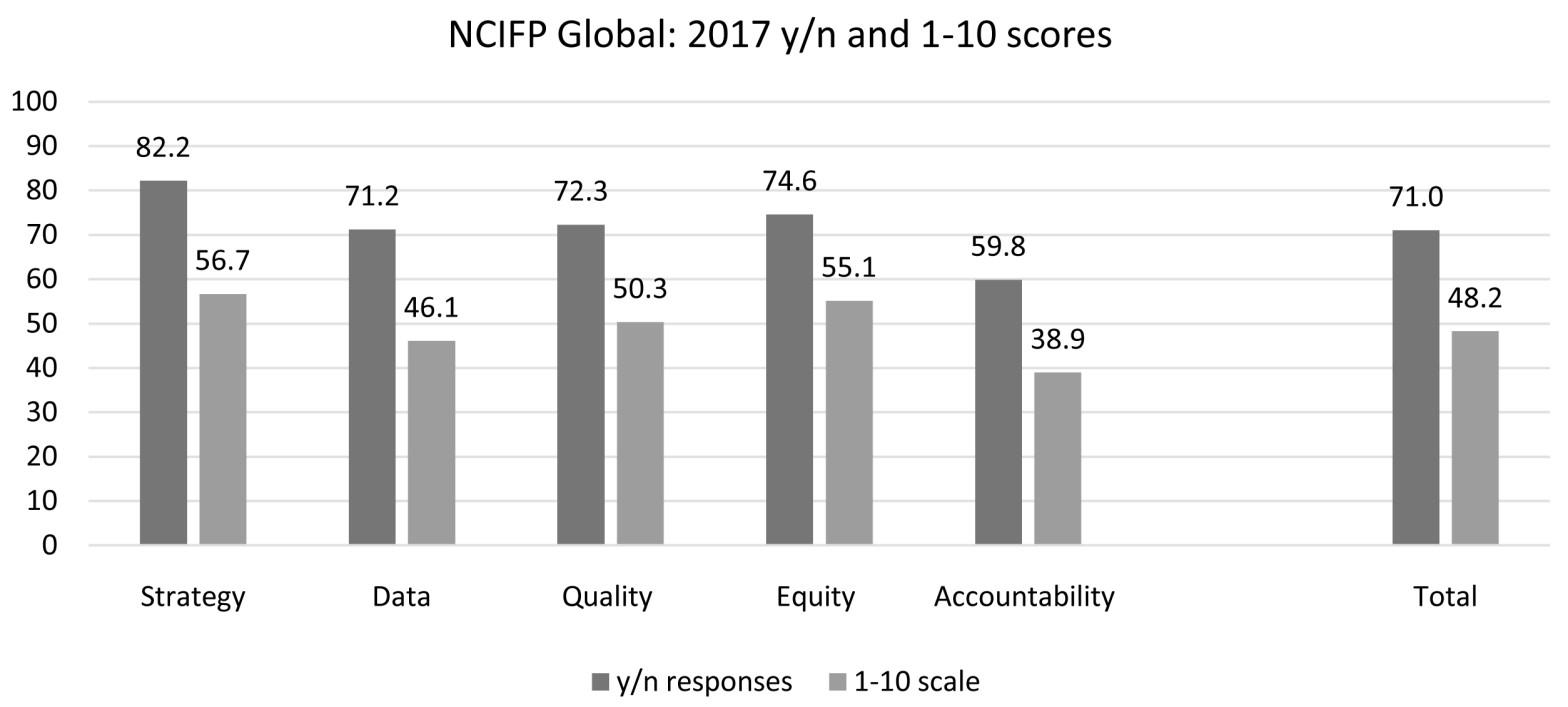
Comparison of Dimension Scores Based on Yes/No Responses and 1-10 Scale Responses.

That permitted a comparison of the two types of ratings: those based only on yes/no replies and those based on the ratings. For the 20 items for which both ratings were used (Table 1 in the *Extended data*), the scores were considerably higher when based on the yes/no replies than when based on the scaled ones ( Figure 7). The total score was higher by 22.8 points (71.0 *versus* 48.2). The differences for the dimensions were not greatly different from each other; in order starting from the Strategy dimension they were 25.5. 25.1, 22.0, 19.5 and 20.9.

Overall, scores based on the 1–10 scale responses tend to be more moderate in range, and more closely grouped across regions. Table 3 provides an overview of the score ranges by response type.

**Table 3.** Minimum Score, Maximum Score, and Range for Individual Item Total Scores, by Response Type (unweighted).

	Yes/No Items (unweighted)	1-10 Items (unweighted)
**Minimum Score**	41.4	30.7
**Maximum Score**	93.4	74.4
**Range**	52.0	43.7

Looking at total scores averaged across all countries, the minimum score for all yes/no items and 1–10 scale items was for “Are violations reviewed on a regular basis?” (41.4 points and 30.7 points, respectively). The maximum score for all yes/no items was for the item “Are FP operating procedures in line with WHO and used for determining areas of need for quality FP improvement?” (93.4), but for the 1–10 items, it was “Extent to which the entire population has ready access to STMs” (74.4 points). The range between the highest-scoring and lowest-scoring yes/no item was 52.0 points, but the range between the highest-scoring and lowest-scoring 1–10 scale item was 43.7 points. Based on these total scores, we see that the 1–10 scale items tend to be more moderate in range than the yes/no items.

For differences across regions ( Table 4), the item with the largest yes/no score difference was “Are indicators for quality of care collected and used for private sector family planning services?” EECA scored lowest across regions (36.7) and SSAF-F scored the highest (78.9), making the regional range 42.2 points. For the 1-10 items, “Extent to which the entire population has ready and easy access to IUD removal” had the widest range across regions (28.3 points), with SSAF-A scoring the lowest (47.9) and EECA scoring the highest (76.2).

**Table 4.** Regional Score Ranges for Individual Items, by Response Type (unweighted).

	Largest Range			Smallest Range		
	Min	Max	Range	Min	Max	Range
**Yes/No Items**	36.7 (EECA)	78.9 (SSAF-F)	42.2	84.4 (EECA)	97.3 (SSAF-F)	12.9
**1–10 Items**	47.9 (SSAF-A)	76.2 (EECA)	28.3	44.8 (SSAF-F)	49.4 (MENA)	4.6

The item with the smallest yes/no score difference across regions was “Are FP operating procedures in line with WHO and used for determining areas of need for quality FP improvement?”, with a difference of only 12.9 points between EECA (84.4) and SSAF-F (97.3). The regional range even smaller among the 1–10 items with a range of only 4.6 for “Are there mechanisms in place to monitor whether or not access to voluntary, non-discriminatory FP information and services is being achieved”, with SSAF-F scoring lowest (44.8) and MENA scoring highest (49.4).

Based on the greatest and smallest range between regions, we notice that scores are much more closely grouped across regions for the 1–10 items compared with the yes/no items.

### Analysis of response rates

To ensure that all responses were informed opinions rather than guesses, respondents were instructed to leave a question blank if they did not know the answer. Non-response therefore serves is an indicator of which components respondents knew less about or which were unclear in the questionnaire wording. That affects the non-response to both yes/no replies and especially to the 1–10 ratings since most respondents choosing a “No” response did not give a 1–10 response.

The response rate refers to the percentage of items in the questionnaire to which respondent gave a reply, not leaving the item blank. This varied both by question and by country. The average response rate across all items and countries was 89.0%, ranging from 61.1% to 99.9% across the 83 countries and from 63.6% to 97.9% across the 35 items.

By question type the average response rate was 91.2% across all yes/no items and 87.8% across all 1–10 scale response items. The ten items with the lowest response rates and the percentage failing to receive a 1–10 rating after a “No” reply are listed in Table 3 of the *Extended data*. Four of the five Accountability items appear in this list, suggesting that the respondents know the least about this component of the family planning program or that the items are the most difficult for respondents to understand. Table 3 of the *Extended data* also shows the ten items with the highest response rates. These were dominated by the scaled scores (nine of 10 items). Four of the items with the highest response rates were related to access or data to monitor access.

### Response rates by region and country

For each country, the average response rate across all 35 questions was calculated and averaged by region. Around the mean rate of 89.5% the regions ranged within a narrow span of 84.3% (MENA) to 90.9% (SSAF-A). The top four regions were clustered at 90%. ( Table 5)

**Table 5.** Average Response Rates by Region.

Region	Mean Response Rate
SSAF-A	90.9
LAC	90.3
SSAF-F	90.2
ASIA	89.5
EECA	88.5
MENA	84.3
**Total**	**89.5**

By country, the range was much greater, from the high 90s down to the low 70s. The ten highest and ten lowest scoring countries are in Table 4 of the *Extended data*, which shows the lack of any regional pattern in the response rates. The high and low countries each fall into a wide variety of regions, so no clear pattern emerges for the determinants of response rates, which differ most by the yes/no *versus* the scaled replies.

### Improvement of response rates between the 2014 and 2017 rounds

The response rates improved between the two rounds of the NCIFP, from 86% to 94% (for countries with data in both rounds). 

**Figure 8.**
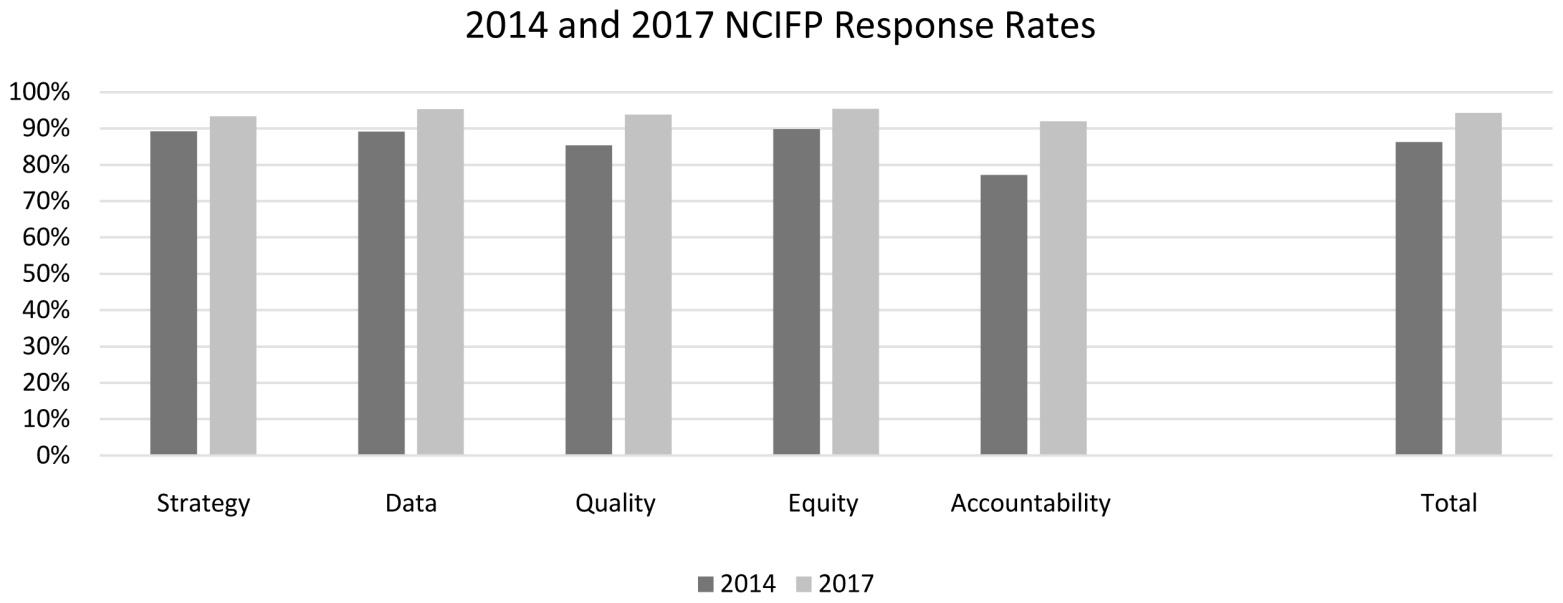
2014 and 2017 NCIFP Response Rates for each Dimension.

Every dimension showed a gain; the largest was for Accountability, which moved up from a very low score in 2014 ( Figure 8). In a sense it had the greatest “room” to grow. Otherwise, the 2017 levels all fell in the 90s.

### Response rate gains by both domain and region

**Figure 9.**
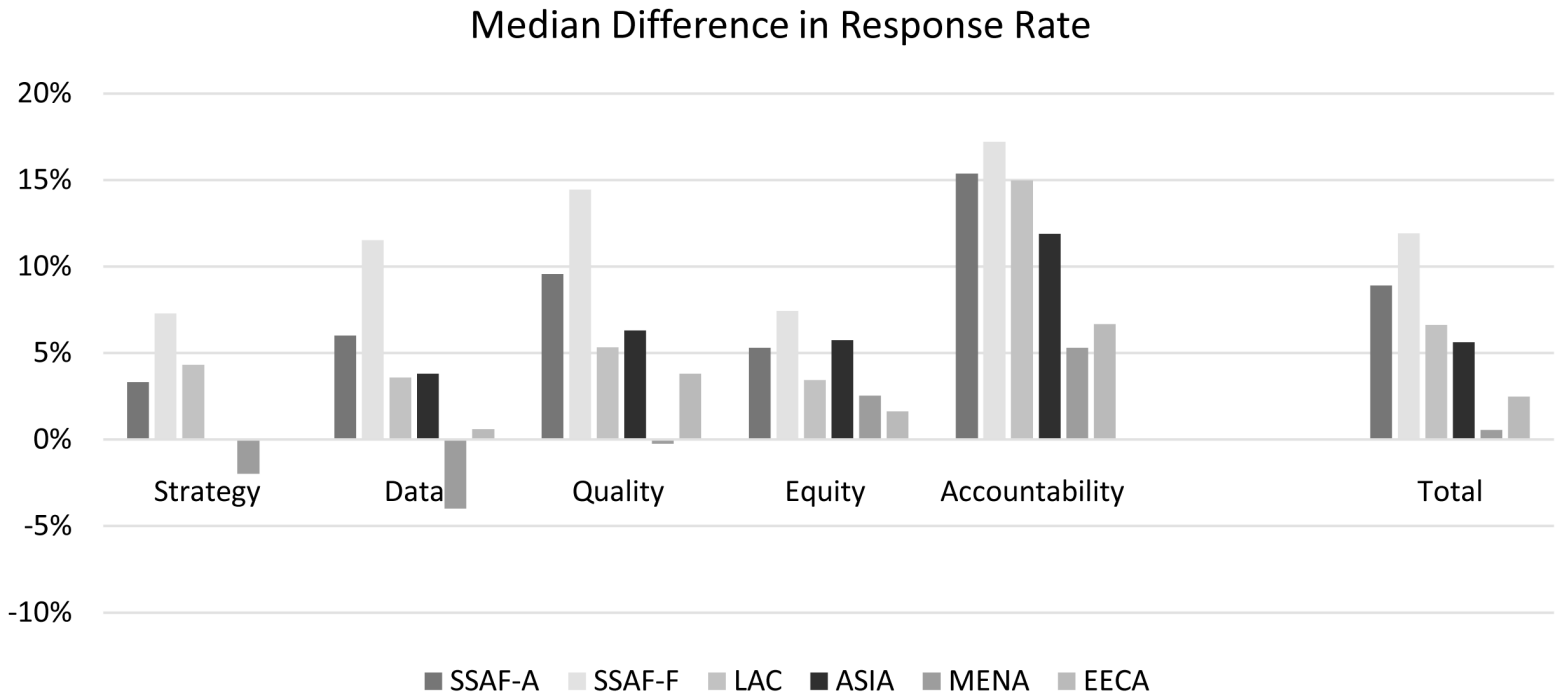
Median Difference in Response Rates from 2014 to 2017.

The regions differed considerably in the degree of gain across the five dimensions. Figure 9 shows the difference between the median scores registered in 2014 and 2017 (for countries with data in both rounds). SSAF-F experienced the greatest gain in every dimension, and SSAF-A was second in three dimensions as well as in the overall total). The rank order for the other regions was generally consistent, especially marked in the Equity dimension. The MENA region’s scores changed the least, and lost ground in two of the dimensions. The Accountability gains were clearly the greatest, starting from the lowest 2014 levels as noted above.

These patterns are suggestive for the policy and programmatic improvements that occurred differentially in the various regions and in the five dimensions. The changes cannot be decomposed by actual improvements and methodological factors, but they point in a positive direction. By country, differential response rates may not be very telling concerning the programmatic efforts as they may partly reflect the selection of respondents and the follow-up efforts of the consultants.

### Accountability dimension

The decomposition question is especially germane to the Accountability dimension, since its average score rose by 20 points. This may be due to actual improvements in some of its five indices. It may also reflect the higher response rates in 2017, which were possibly selective by respondent type, introducing better levels of knowledge that favored higher scores. Another consideration is the change in the questionnaire, asking for a 1–10 rating in 2017 in addition to the yes/no rating; however as noted above, the Results section above is based on analyses using just the 2014 approach, for comparability across rounds. 

To explore reasons for the large increase in part of the Accountability score, we looked at the scores (yes, no, or no responses, adding to 100%) across all replies in Table 6 for each of the five Accountability items (again only for countries that completed the NCIFP in both 2014 and 2017; also, Table 6 pertains only to the yes/no responses in since the 1–10 scale response was not an option for any of the Accountability items in 2014).

**Table 6.** Yes/No Scores and Non-Response (NR) for Accountability Items in 2014 and 2017.

	2014 NCIFP (N=955)			2017 NCIFP (N=1,006)		
Accountability item	% Yes	% No	% NR	% Yes	% No	% NR
Are there mechanisms in place at the national, subnational, and facility level to monitor whether or not access to voluntary, non-discriminatory FP information and services is being achieved?	39	43	18	68	26	6
Does the government have mechanisms in place for reporting instances of denial of services on non-medical grounds (age, marital status, ability to pay), or coercion (including inappropriate use of incentives to clients or providers)?	20	55	26	40	50	10
Are violations reviewed on a regular basis?	19	55	26	35	51	14
Are there mechanisms in place at the facility level to solicit and use feedback from clients?	32	45	23	62	32	6
Is there a system in place that encourages dialogue and communication between users and service providers/health officials about service availability, accessibility, acceptability, and quality? (The system for dialogue and communication can include interviews after clinic visits, regular community forums, joint quality improvement systems, or other interactive sessions.)	39	39	22	68	27	4

Frequency of “Yes” replies rose markedly in 2017, in every item, gaining 29, 20, 16, 30, and 29 percentage points in order across the five indices, and by 25 percentage points overall. These increases came partly at the expense of the frequency of “No” replies, which declined by 17, 5, 4, 13, 12, percentage points respectively, and by 10 percentage points overall. They also came partly at the expense of the “no response” replies, which declined by 12, 15, 12, 17, and 18 percentage points in order across the five items, and by 15 percentage points overall. These shifts clearly suggest real gains in effort, and the possibility that the large increase in replies were selective in giving higher ratings, possibility due to a firmer grasp of the nature of the program and of the indices during the three years.

That raises the question of the kinds of training provided in that period and the stress given to all dimensions, but in particular to the Accountability dimension, which was scored lowest in 2014. Actions taken at and after the 2012 London Summit on Family Planning have been reviewed by Hardee ^11^.

## Discussion

The NCIFP is a measurement tool to help capture the enabling environment in which family planning programs are implemented. It builds on the earlier FPE series to capture information on features omitted in the past, particularly issues related to rights (quality and accountability) and equity. Results of the 2017 round of the NCIFP show improvements in policies and program implementation across all five dimensions of effort: Strategy, Data, Quality, Equity, and Accountability. In both 2014 and 2017, Strategy was the highest scoring dimension and Accountability the lowest.

Results are presented globally, by region and by country, to assist with policy judgements and resource allocations. At the country level, decision-makers can review the scores to identify specific areas for improvement. Patterns in scores across regions are quite consistent, indicating that some program features are intrinsically more difficult to address than others. For example, most regions scored well on items related to defined objectives, standard operating procedures, and quality control, but lower on items related to access to long-acting methods, to monitoring denial of services, and to reviews of violations related to coercion and denials of services.

Scores that improved over time are important, although they may have partly reflected methodological changes in the questionnaire in 2017. The Accountability dimension improved the most; though it was the lowest scoring dimension in both rounds, it saw the largest increase over time. This increase is likely due to higher scores as the response rates rose markedly, with the additional respondents giving higher ratings, as well as to higher estimates given by other respondents. Such differential changes by dimension and across the 35 indicators give further information for policy and programmatic adjustments.

The association between contraceptive use and the total NCIFP score was positive among countries in sub-Saharan Africa, but very weak for countries in other regions. That pattern existed not just for the total score but also for each of the five dimension scores. A moderate correlation exists between the NCIFP for access to LAPMs and the percent of modern users using them, suggesting that efforts to improve access may impact use.

### Limitations

The results in this report are limited in several respects, partly for the methodological issues discussed. The time trends shown are only for countries participating in both rounds, and the sets of respondents between rounds in each country varied to some extent. The degrees of respondent biases in the scoring of program features are unknown; the use of four types of respondents in each country acted as a partial protection against them, as did the consistency checks in the centralized data processing. Finally, the scores rely on the judgments of program observers with a necessary element of subjectivity; that is the price paid for obtaining scores for most developing countries on a standard questionnaire, at a single point in time.

## Conclusion

The NCIFP is the first large-scale survey to cover both the previous topics on program features and also the important ones of equity and accountability. Additional research is needed to gain a better understanding of the five dimensions of Strategy, Data, Quality, Equity, and Accountability. As more rounds of the NCIFP are completed, it will be possible to trace more clearly how changes in the NCIFP move from their current levels, and how they relate to other family planning indicators.

## Data availability

Zenodo: Underlying data for ‘The National Composite Index for Family Planning (NCIFP): results and methodological issues’.

Raw data, tables and figures: https://doi.org/10.5281/zenodo.5735184 ^12^

Extended data tables: https://doi.org/10.5281/zenodo.5735404 ^13^

This project contains the following underlying data:

- The National Composite Index for Family Planning (NCIFP): results and methodological issues (The file includes raw data, as well as a tab for each table and figure presented in this paper).
- The National Composite Index for Family Planning (NCIFP): extended data (The file contains supplementary tables to accompany this paper).

Data are available under the terms of the Creative Commons Attribution 4.0 International.

## Consent

Written informed consent was obtained to take part in this research and all data has been anonymized.
