# Supplementary material for: The National Composite Index for Family Planning (NCIFP): Results and Methodological Issues
Source: Gates Open Res. 2022 Jan 7;6:1. [Version 1] doi: 10.12688/gatesopenres.13464.1 (PMC10169564; doi:10.12688/gatesopenres.13464.1)
Supplement: Peer review history

# Peer review: Reviewer response for version 1

**DOI:** 10.21956/gatesopenres.14722.r33117

**Published:** 2023-05-26

**Authors:** Frida Gyllenberg

**Affiliations:** 1 City of Vantaa, Vantaa, Uusimaa, Finland; 2 University of Helsinki, Helsinki, Finland

This paper describes the introduction of a novel tool for measurement of family planning efforts. The National Composite Index for Family Planning (NCIFP) was added to the Family Planning Effort Index (FPE) in 2014 to capture areas not covered by the FPE. The authors present results from 2014 and 2017, when the questionnaire was completed in 89 and 84 countries, respectively. The tool was adjusted before the 2017 round, and presents a comprehensive approach to measure family planning efforts.

 The paper is well written and clearly defines the methodological considerations regarding the substantial changes made to the questionnaire between 2014 and 2017. Nevertheless, the main area of concern still is that a large portion of the results presented refers to the change between 2014 and 2017, and thus refers more to the FPE than to the NCIFP. I think it would be important to emphasize this, perhaps already in the abstract (and Title?), that a large part of the results presented refers to the FPE as the two rounds of NCIFP cannot be compared.

 In addition, I have some comments and questions that I believe could improve the paper if addressed:

1. In the Methods section, second last paragraph: Do you weight the average scores by the number of women aged 15 – 19? Why this age group and not e.g. 15-49?
2. In the Results section, I would have appreciated a legend over how to interpret the scores. For example, is the average score of 64 a good or poor result? How high would the score be in a country with e.g., easy access to family planning services of good quality? Figure 1 e.g., would be easier to interpret with information on what a certain score relates to.
3. Writing out the names of the regions in figure legends could increase readability.
4. Regarding trends and changes between 2014 and 2017, the authors state that trends can only be assessed for FPE items, as there were changes in the NCIFP questions between 2014 and 2017. However, Figure 5 presents changes over time also for items not in the FPE. Are these comparisons then made over items not worded the same? I think this needs to be clarified in the text. In addition, I think it could be good to emphasize, perhaps already in the abstract, that a large part of the results presented refers to the FPE as the two rounds of NCIFP cannot be compared.
5. In Figure 2, the “Increase” bar is unnecessary. The change can be seen from the “year” bars, and the absolute numbers and percentages are written in the text. If the authors want to highlight the increase, maybe this could be noted above each pair of “year” bars?
6. Throughout the results section, please clarify why either weighted or unweighted results are presented. Why is it better to compare unweighted results in Figure 2 but weighted results in Figure 4?
7. Please define mCPR at its first appearance.
8. In the methods section, the authors name “correlation approaches” as an analytic technique. In the results section and in Table 2 this correlation is presented as *r*=… Please specify how correlation was assessed in both text and Table legend to increase readability. If the *r *stands for the Pearson’s correlation coefficient, could you provide a p-value according to APA standards?
9. Make sure the year of the results is clearly stated for each figure/table (e.g., Table 3-5).

Is the work clearly and accurately presented and does it cite the current literature?

Yes

If applicable, is the statistical analysis and its interpretation appropriate?

Yes

Are all the source data underlying the results available to ensure full reproducibility?

Yes

Is the study design appropriate and is the work technically sound?

Yes

Are the conclusions drawn adequately supported by the results?

Yes

Are sufficient details of methods and analysis provided to allow replication by others?

Partly

Reviewer Expertise:

Family Planning policies: implementation, evaluation.

I confirm that I have read this submission and believe that I have an appropriate level of expertise to confirm that it is of an acceptable scientific standard, however I have significant reservations, as outlined above.

# Peer review: Reviewer response for version 1

**DOI:** 10.21956/gatesopenres.14722.r32979

**Published:** 2023-05-09

**Authors:** Leontine Alkema

**Affiliations:** 1 Department of Biostatistics and Epidemiology, University of Massachusetts Amherst, Amherst, MA, USA

This paper provides a nice introduction to the NCIFP and includes various descriptive analyses based on the 2017 round, documenting differences between countries, changes over time, and assessments of the various dimensions. Limitations associated with the data collection and measurement are adequately discussed.

 My only suggestion relates to the analysis of the association between the score and mCPR, assessed using country data for 2017. Given the many factors affecting mCPR in an across-country analysis, results from a regression of mCPR on just the score are difficult to interpret.

 It seems to me that this paper has sufficient content without it so I would suggest removing it from this paper. If some analysis is of interest, I would suggest considering within-country change between 2014 and 2017, and checking association of that with the (changes in) score while accounting for other variables, preferably also considering possible regional variation in relationships, non-linear relationships, and interaction terms.

Is the work clearly and accurately presented and does it cite the current literature?

Yes

If applicable, is the statistical analysis and its interpretation appropriate?

Partly

Are all the source data underlying the results available to ensure full reproducibility?

Yes

Is the study design appropriate and is the work technically sound?

Yes

Are the conclusions drawn adequately supported by the results?

Yes

Are sufficient details of methods and analysis provided to allow replication by others?

Yes

Reviewer Expertise:

Statistical modeling for global health indictors, in particular for family planning, reproductive, maternal, and child health.

I confirm that I have read this submission and believe that I have an appropriate level of expertise to confirm that it is of an acceptable scientific standard.

# Peer review: Reviewer response for version 1

**DOI:** 10.21956/gatesopenres.14722.r31622

**Published:** 2022-02-01

**Authors:** Ellen H. Starbird, Amani Selim

**Affiliations:** 1 Office of Population and Reproductive Health, United States Agency for International Development (USAID), Washington, DC, USA; 2 USAID, Office of Population and Reproductive Health, Washington, DC, USA

This paper reports on results from key informant interviews on the strength of various aspects of family planning programs in 89 countries (2014), and 84 countries (2017). It is the latest in a series of such analyses going back to 1972 and expands on prior work by incorporating the NCIFP. The NCIFP adds questions on rights (quality and accountability) and equity that were not part of the earlier Family Planning Effort scores. The paper is scientifically sound and acknowledges the drawbacks of key informant interviews. The paper is principally descriptive, presenting an analysis of the results without making policy or program recommendations. 

 The inclusion of the accountability questions bring attention to accountability as an important component of FP program progress and provide a baseline for future assessments of accountability using this kind of instrument. It is disappointing, however, that the construction of the NCIFP did not go further and include questions to assess increases in, diversity in sources of, or efficient use of financing or questions around the extent to which FP programs are viewed as contributing to transformation in social and gender norms. We hope the index will continue to evolve in these areas.  

 While we endorse indexing of the paper in its current form, we have the following suggestions for the authors' consideration:

1. The development of the NCIFP was a response to the focus areas of FP2020. As noted above, however, the index does not address all of those areas. The authors might want to say more about why, any plans for expanding the index, and the value of doing so.
2. Results: The results section notes that scores were highest in Africa. Given that FP programs in AFR are generally less well-established than those in LAC or Asia, this is a surprising finding. What hypotheses do the authors have for this result?
3. Methodological discussion. Yes/no responses vs the 1-10 scale: The authors find a larger range in scores using the yes/no question formulation vs. the 1-10 scale. They note that a score of "1" on the 1-10 scale could be interpreted as a "no." Was consideration given to adjusting "yeses" to "nos" where a "yes" score was accompanied by a "1" ranking on the 1-10 scale? Does the range become more narrow?
4. The paper notes a weak relationship between levels of contraceptive use and the NCIFP score outside of Africa, a somewhat surprising result. Is this result consistent across earlier iterations of the FPE scores or a new result now? More discussion of this finding would be useful.
5. What is the hypothesis for analysing the relationship of contraceptive use by youth to selected NCIFP indicators? Comparing the results of the NCIFP data collected from key informants to DHS data for sexually active youth (15-24 years) needs more analysis or matching with other youth related survey data. 

Is the work clearly and accurately presented and does it cite the current literature?

Yes

If applicable, is the statistical analysis and its interpretation appropriate?

Yes

Are all the source data underlying the results available to ensure full reproducibility?

Yes

Is the study design appropriate and is the work technically sound?

Yes

Are the conclusions drawn adequately supported by the results?

Yes

Are sufficient details of methods and analysis provided to allow replication by others?

Yes

Reviewer Expertise:

Starbird:family planning policies and program implementation; Selim: FP program monitoring and evaluation

We confirm that we have read this submission and believe that we have an appropriate level of expertise to confirm that it is of an acceptable scientific standard.
